# The Scandinavian Physiological Society in 100 years—Looking ahead

**DOI:** 10.14814/phy2.70181

**Published:** 2025-01-04

**Authors:** Helle Prætorius, Vladimir Matchkov, Nanna Brinch, Tobias Wang

**Affiliations:** ^1^ The Executive Committee the Scandinavian Physiological Society Aarhus University Aarhus Denmark

The Nordic countries have a strong tradition in physiological research with an early influence that reached far beyond the regional borders, and the Scandinavian Physiological Society (SPS) continues to play an essential role in physiology in northern Europe. We, therefore, look very much forward to celebrating our centenary and illustrious tradition in 2025. Based in the Nordic fellowship of countries, the Scandinavian Physiological Society engages with societies and researchers around the world with the vision to *unite researchers in physiology and other life sciences*—a vision that has no borders.

To realize the vision of the Scandinavian Physiological Society, we have pledged to collaborate on our annual meetings to ensure a strong platform that enables the community to present science of excellent quality and to engage in network activities that often lead to new collaborations. In that spirit, and with our wish to strengthen international collaboration, we seek to further improve the quality of physiology conferences, and we are pleased that our next annual meeting will be part of the upcoming congress of the International Union of Physiological Sciences (IUPS) in Frankfurt in September 2025. This strategy has been applied with great success over the past years. In 2023, we held our annual meeting Physiology in Focus in Tallinn (Estonia) in collaboration with the Baltic Physiological Societies, and earlier this year, we joined forces with the Physiological Society when co‐organizing Physiology in Focus 2024 meeting in Newcastle upon Tyne. These important network activities plan to minimize environmental footprint by organizing the meetings in a European setting to reduce the overall travel miles and encourage use of public ground transportation via a flexible funding scale.

The trademark of the Scandinavian Physiological Society at these joint conferences is the active involvement of our Special Interest Groups (SIGs) in the pre‐meetings that typically take place on the day immediately before the official opening of the conference. The SIGs are run in a bottom‐up manner with the active engagement of early‐career researchers and are supported by an annual budget from the Scandinavian Physiological Society. Our only stipulation is that each SIG arranges a symposium during the pre‐meeting, and our SIGs currently represent independent groupings in cardiac, comparative, vascular, renal, skeletal muscle physiology, and neuroscience. The SIGs are also encouraged to arrange smaller independent events, such as focused workshops or training sessions. Generally, the efforts of the SIGs are highly valued by the participants of our combined annual meetings—and their activities are alongside the name “Physiology in Focus,” an easily recognizable element of the meetings the SPS participates in.

As a recent initiative, the Scandinavian Physiological Society has established two named plenary lectures at our annual meetings. The most prestigious award is the *Christian Bohr lecture*, bestowed as a Lifetime Achievement Award to an individual with remarkable and pioneering research achievements within physiology. The *Ulf von Euler Lecture* is awarded for scientific excellence. In addition, SPS has funded the *August Krogh lecture* at the meeting of the International Union of Physiological Sciences since 1986.

A large part of the Scandinavian Physiological Society's support for experimental science goes through the activities of *Acta Physiologica*, of which SPS has been proud owner since 1940. In line with the broad engagement in physiology in the Nordic countries, *Acta Physiologica*, as a high‐end society journal, invites manuscripts in all areas of experimental life sciences. Established in 1889 as the *Skandinavisches Archiv für Physiologie*, *Acta Physiologica* stands on a long tradition as a recognized international forum where physiologists publish their scientific findings. Since the Scandinavian Physiological Society took ownership, we continue to be actively engaged in keeping the journal relevant for the ever‐changing publishing demand from our authors. Under the abbreviated name *Acta Physiologica* (since 2006), the journal has managed to become one of the high‐standing journals among society journals in physiology. In a time challenged by the fictional and the AI‐generated manuscripts as well as the rapid emergence of non‐society, profit‐oriented journals, we believe that rigorous peer review and quality control are of paramount importance. Society‐owned journals hold the key to maintaining the solid academic virtues that have brought progress and enlightenment while returning profits to the scientific community. Currently, the physiological societies provide a rich portfolio of journals to suit any publishing need of experimental scientists. From this platform, we are happy to constitute one of the strong columns for publishing in society journals. The Scandinavian Physiological Society ensures that the surplus from our publishing activities is funneled back into science by supporting our SIGs, scientific meetings, and the ability of our members to attend them, and we work to strengthen the bonds between the Society and Acta Physiologica even further in the upcoming years.

With our choice of a new Editor‐in‐Chief for *Acta Physiologica* for 2025, the Scandinavian Physiological Society emphasizes physiology as a stronghold for both biological and medical sciences. We want to underscore the importance of physiology in adaptation to new climate and biodiversity challenges and believe that the understanding of the physiological mechanism is crucial to understanding the physiology of healthy organisms and its responses during disease and clinical manifestations. We therefore seek to emphasize an integrative approach, where mechanisms are disclosed at lower levels of biological organisms, while their functional consequences are revealed at the organismal level. Through this, we build on our strong history to assure consistency through constant renewal that is part of the *Acta Physiologica* brand.

The Scandinavian Physiological Society and *Acta Physiologica* have, from the very beginning, endorsed and supported Physiological Reports. We believe *Physiological Reports* represents an important outlet for sound and good physiological studies and find the automatic transfer among journals a very sensible use of the resources spent by reviewers and a great help to the authors. It is our privilege, therefore, to congratulate the 10‐year anniversary of *Physiological Reports* and rejoice in the success of creating a platform for an open‐access journal publishing solid peer‐reviewed physiology. *Acta Physiologica* is very pleased to continue our referral manuscripts to *Physiological Reports*, and it is our ambition to facilitate and increase the overall collaboration between society‐owned physiology journals.

## FUNDING INFORMATION

No funding was involved in the preparation of this editorial.

## ETHICS STATEMENT

The authors are members of the executive committee of the Scandinavian Physiological Society. Helle Prætorius Øhrwald (HPO) is the president, Vladimir M. Matchkov is the secretary general, Nanna Brinch is the administrative coordinator and communicator, and Tobias Wang is the executive officer of the Scandinavian Physiological Society. HPO is currently a member of the Editorial Board of Physiological Reports and was not involved in reviewing or making decisions for this manuscript.

